# B-cell Activating Factor Polymorphisms in Rheumatoid Arthritis-Associated Atherosclerosis

**DOI:** 10.31138/mjr.32.2.179

**Published:** 2021-06-30

**Authors:** Nikolaos Kintrilis, Adrianos Nezos, Evangelos Theodorou, Michalis Koutsilieris, Clio P. Mavragani

**Affiliations:** 1 Department of Physiology, School of Medicine, National and Kapodistrian University of Athens, Athens, Greece; 2 Second Internal Medicine Unit, 401 General Military Hospital of Athens, Athens, Greece; 3 Rheumatology Unit, 251 Air Force General Hospital, Athens, Greece; 4 Rheumatology Outpatient Unit, Department of Pathophysiology, School of Medicine, National and Kapodistrian University of Athens, Laikon Hospital, Athens, Greece

**Keywords:** Rheumatoid arthritis, atherosclerosis, cardiovascular disease, B-cell activating factor

## Abstract

Rheumatoid Arthritis (RA) is a common chronic inflammatory disorder affecting 0,5–1% of the population, characterised by intense cellular activation and inflammation in the affected joints ultimately leading to bone and cartilage destruction. Cardiovascular disease is the leading cause of death among patients suffering from RA, with chronic inflammation and genetic background emerging as major predisposing factors. Although the pathogenetic events leading to an increased rate of atherosclerosis in the affected group are not precisely described, several genetic variations have been suggested as possible mediators of this process. The aim of the current research proposal is to investigate the role of B-cell activating factor (BAFF) variants in the pathogenesis of RA-related atherosclerosis. Stored DNA samples from the Biobank in the Department of Physiology of the Medical School of the University of Athens from RA individuals and healthy controls will be analysed for polymorphisms of B-cell activating factor (BAFF) by polymeric chain reaction (PCR) based assays. Detection of plaque formation and calculation of the mean intima media thickness (mIMT) of the vessel wall will be performed in RA patients by using carotid and femoral artery ultrasonography. Complete personal and family history, biochemical and serological markers will be obtained from the RA group and associated with the genetic and IMT data. The results will be compared across the different subgroups in order to determine whether any particular genetic variants can act as prognostic markers for RA-related cardiovascular disease giving eventually new insights to atherosclerotic processes in the context of chronic inflammatory diseases. Such a result would invariably lead to a possible new treatment approach and/or prevention method to benefit this group of patients.

## BACKGROUND/OBJECTIVE

Rheumatoid Arthritis (RA) is an autoimmune disorder that mainly affects an individual’s joints, as well as the underlying bone and cartilage, presenting with warm, red, swollen, stiff and painful joints, especially after periods of rest.^1^ In 2015, RA affect about 24.5 million people worldwide, equal to 0.5–1% of adults in the developed world. The disease mainly affects women; female to male ratio is about 3:1 to 5:1.^2,3^ The incidence of the disease rises with age, with the most common age group affected being middle-aged females of 40 to 50 years of age.^4^ Physiologically, RA manifests as intense cellular activation resulting to autoimmunity and formation of immune complexes in affected sites. The disease usually presents bilaterally on hands and wrists, while other common manifestations include fever, exhaustion, osteoporosis, interstitial lung disease, mental health problems and complications such as frequent infections.^5,6^ Although the exact aetiology of the disease remains unknown, some genetic and environmental factors –such as smoking-have been pinpointed so far.^7^

B-cells maintain a plethora of potential key pathogenetic roles, among which secreting inflammatory cytokines and producing Rheumatoid Factor (RF) as well as anti-citrullinated protein antibodies (ACPAs) in peripheral blood and synovial tissue.^8^ Furthermore, B-cells can contribute to RA pathogenesis via antibody-independent pathways, mainly via acting as antigen-presenting cells or leading to modulation of T- and dendritic cell functions.^9^ B-cell activating factor (BAFF), otherwise known as tumour necrosis factor ligand superfamily member 13B is a protein encoded in the human genome by the TNFSF13B gene, located in the q area of human chromosome 13. In essence, BAFF is a cytokine expressed in B-cell lineage cells, which not only acts as a B cell family activating factor, but has also been shown to affect the differentiation and proliferation of B cells. BAFF is a 285-amino acid long peptide glycoprotein, expressed a transmembrane protein on various cell types, such as dendrite cells, monocytes, and marrow stromal cells. BAFF is the natural ligand of three tumour necrosis factors, namely BAFF-R, TACI (transmembrane activator and calcium modulator and cyclophilin ligand interactor) and BCMA (B-cell maturation antigen), all of which can bind to it, with various levels of affinity. All receptors are expressed mainly on mature B-lymphocytes and the level of expression corresponds to B-cell maturity.^10,11,12^ Atherosclerosis is a state in which the formation of plaque inside the human arteries leads to narrowing of their diameter via remodelling of the artery wall, with accumulation of fatty substances under the endothelium. Although this may be asymptomatic in the beginning, with the progression of time, the restricted blood flow and the following restriction to tissue oxygen provision can lead to coronary artery disease, stroke, renal failure, or other problems, depending on which arteries are affected. Atherogenesis is a diffuse malfunction of the arterial wall, characterized by activation of inflammation procedures, oxidative stress, altered metabolism of lipids, lipoproteins and glucose, thrombosis procedures and mutations. Multiple risk factors have been associated with the creation of plaque within the arterial walls, both modifiable (diabetes and metabolic syndrome, elevated cholesterol levels, smoking, hypertension) and non-modifiable (age, sex, family history and genetic factors). Research continues to pinpoint more factors to which elevated atherosclerotic levels could be attributed, such as thrombophilia, diet, sedentary lifestyle, chronic stress, and personality type.^13^

## AIM OF THE STUDY

To investigate the role of variations of the BAFF gene, in the pathogenesis of RA-related atherosclerosis.

## STUDY PARTICIPANTS

In our Biobank in the Department of Physiology of the Medical School of the University of Athens, we have collected 180 RA patient peripheral blood and serum samples, with complete personal and medical history, and an equal number of healthy controls (HC), after informed consent was obtained. These patients are followed up in the Outpatient Rheumatology Clinic, Department of Pathophysiology and General Hospital of Athens “G. Gennimatas” and fulfilled the American College of Rheumatology classification criteria for RA.^14^ Among the RA patients, RA and ACPA status are tracked and a stratified analysis will be performed to determine possible correlation of atherosclerosis levels and seropositivity. Exclusion criteria for both groups are age younger than 18 years old, known pregnancy at the time of inclusion in the study, and serious renal impairment, defined as chronic kidney disease (CKD) stages IV and V, or glomerular filtration rate (GFR) less than 30 ml/min. RA and HC groups are of Caucasian origin, age- and gender-matched. Informed consent for participation in the study was obtained from all subjects and the study has been approved by the Ethics Committee of University of Athens and Laikon General Hospital of Athens.

## STUDY PROTOCOL

The main expected outcome is higher rates of subclinical atherosclerosis among patients with specific BAFF polymorphisms, as assessed by ultrasound imaging methods. In more detail, carotid and femoral artery ultra-sound will be performed in all RA patients. Intima Media Thickness (IMT) scores will be measured across various sites and the presence of intra-arterial plaque as markers of subclinical atherosclerosis will be noted. Genomic DNA will be extracted from the peripheral blood samples of all study participants and common polymorphisms of the B-cell activating factor (BAFF) will be evaluated by PCR based assays. Furthermore, quantitative determination of peripheral blood BAFF mRNA transcripts and serum protein levels will be performed by Real-Time PCR and ELISA, respectively. Statistical analysis will be performed by SNPStats and SPSS software. Simple descriptive analyses of proportion will be used for categorical variables (plaque presence, vascular stenosis) and mean with standard deviation for continuous variables (IMT). Chi-square and Mann-Whitney tests will be used for the comparison of categorical and continuous variables, respectively, between patients with or without arterial plaque and/or arterial wall thickening. Multivariate models will be used for variables resulting from univariate analysis for independent correlation of SNP variants with subclinical atherosclerosis levels.

## SIGNIFICANCE

Recent studies have brought to light an independent correlation between BAFF levels and subclinical atherosclerosis in patients suffering from SLE and highlighted the contribution of MTHFR gene variants in lupus-related subclinical atherosclerosis.^15,16^ Although the link between RA and cardiovascular mortality development had first been appreciated a while ago, the underlying pathogenetic events mediating the process remain largely unresolved. Our findings could help us define the prognostic value of novel biomarkers for RA-related atherosclerosis and propose new treatment approaches (ie, targeted therapies) or preventive measures and strategies that may benefit these patients.
